# Structure, Surface Morphology, Chemical Composition, and Sensing Properties of SnO_2_ Thin Films in an Oxidizing Atmosphere

**DOI:** 10.3390/s21175741

**Published:** 2021-08-26

**Authors:** Weronika Izydorczyk, Jacek Izydorczyk

**Affiliations:** Department of Telecommunications and Teleinformatic, Silesian University of Technology, Akademicka 16, 44-100 Gliwice, Poland; jacek.izydorczyk@polsl.pl

**Keywords:** tin dioxide, DC reactive magnetron sputtering, RGTO technique, surface morphology, X-ray diffraction (XRD), X-ray photoelectron spectroscopy (XPS), oxygen adsorption

## Abstract

We conducted experiments on SnO_2_ thin layers to determine the dependencies between the stoichiometry, electrochemical properties, and structure. This study focused on features such as the film structure, working temperature, layer chemistry, and atmosphere composition, which play a crucial role in the oxygen sensor operation. We tested two kinds of resistive SnO_2_ layers, which had different grain dimensions, thicknesses, and morphologies. Gas-sensing layers fabricated by two methods, a rheotaxial growth and thermal oxidation (RGTO) process and DC reactive magnetron sputtering, were examined in this work. The crystalline structure of SnO_2_ films synthesized by both methods was characterized using XRD, and the crystallite size was determined from XRD and AFM measurements. Chemical characterization was carried out using X-ray photoelectron (XPS) and Auger electron (AES) spectroscopy for the surface and the near-surface film region (in-depth profiles). We investigated the layer resistance for different oxygen concentrations within a range of 1–4%, in a nitrogen atmosphere. Additionally, resistance measurements within a temperature range of 423–623 K were analyzed. We assumed a flat grain geometry in theoretical modeling for comparing the results of measurements with the calculated results.

## 1. Introduction

The surface deposition of semiconductor metal oxides, such as SnO_2_, TiO_2_, ZnO, In_2_O_3_, and WO_3_, is used to create sensitive films for gas sensors [1,2,3,4,5,6]. Sensors based on tin dioxide are widely researched due to their application in toxic gas concentration monitoring, mainly in industry and areas with polluted atmospheres. Oxygen on top of such surface layers is an essential part of the system because it is highly reactive [7,8,9,10,11]. Oxygen absorbed on a layer’s surface reduces its conductance, emergence, and rises its work function (in the range of 423–723 K) [12,13]. On the other hand, the control of oxygen concentration is crucial in cooling, food production [14,15], gardening [16], diagnostics [17], and alarm devices such as oxygen monitors for the atmosphere or water [18,19], where improper oxygen concentrations lead to underwater corrosion phenomena [20]. The SnO_2_ layer properties are crucial in determining the interaction of gas with the surface and, consequently, developing new sensors. Typically, sensors are able to work in the temperature range of 420–720 K at 1 atm pressure in an atmosphere with high concentrations of oxygen. Under such conditions, surface oxygen frequently reacts with atmospheric gas.

Additionally, different methods of creating gas-sensing layers and further processing, for example, aging in a humid atmosphere [21], may influence the response range [22,23,24,25,26,27]. Therefore, the development of highly sensitive and selective sensors depends on understanding the absorption processes and chemistry of compounds inside the sensor layer [8]. Some elements, such as Pt, Pd, Au, and Sb, are frequently added to the sensor material in small quantities to improve sensor selectivity and sensitivity [1,8,28,29,30]. However, the sensitivity of tin dioxide-based sensors depends primarily on the amount of oxygen removed from the SnO_2_ oxide lattice. Moreover, control over surface defects, sensor material additives, and the extended surface–gas interaction are crucial [7,8,31]. Previous studies have shown that the surface sensitivity of a thin-film sensor to the atmosphere depends on the smoothness, purity, and temperature of the tin dioxide substrate, as well as on the properties of the tin dioxide substrate, crystal size, temperature, and humidity of the active surface layer [32,33]. Previous research has focused mainly on oxygen and synthetic air interactions with thick films [34,35], whereas thin films have been investigated in fewer papers [36,37,38].

In this paper, we present comparative studies of SnO_2_ layers produced using two different methods of fabrication. Tin dioxide films were deposited on quartz substrate using the DC reactive magnetron sputtering method (MS-SnO_2_ films) and on alundum ceramic using a rheotaxial growth and thermal oxidation process (RGTO-SnO_2_ films). These layers may be useful for the detection of oxidizing (e.g., NO_2_ and O_2_) and reducing (e.g., H_2_) gases [39,40,41]. Because the gas molecules interact only with the surface atomic layers, the accurate characterization of these layers in terms of morphology and surface structure is essential. In this paper, we present our results on the structure and surface morphology, chemical composition, and sensing properties of both types of synthesized SnO_2_ thin films. The SnO_2_ thin films were characterized in detail through XRD, SEM, AFM, AES, and XPS studies. In addition to data on the composition of the surface layers, we obtained information about the chemical environment or oxidation state of a given element. We studied the effect of annealing on the sample resistance changes in an oxygen atmosphere as a function of temperature to determine relationships between the surface morphology and conductance response. We then compared the obtained measurement results with theoretical calculations, where a flat grain geometry was assumed in theoretical modeling.

## 2. Materials and Methods

The dependence of structure and chemistry on the electronic properties of SnO_2_ thin layers was determined by analyzing SnO_2_ surface structure and topography and carrying out gas tests. The morphology and topography of the SnO_2_ surface were determined using SEM and AFM measurements. AES, XPS, and XRD were used to determine the specimen composition and crystallinity.

### 2.1. Production of SnO_2_ Thin Layers

The SnO_2_ thin films investigated in this paper were grown by either RGTO or magnetron sputtering. In the case of RGTO, tin droplets of 99.99% purity were deposited on alumina substrate and heated to a temperature range of 528–543 K using vacuum thermal evaporation (p = 10^−3^ Pa). This was followed by thermal oxidation of the tin layers in an oxygen-containing atmosphere at 973 K to produce SnO_2_ [33,40]. The proper thermal environment is crucial to avoid incomplete oxidation of the metal droplets, which may lead to unwanted sensor response drift [41]. A detailed description of sensor materials prepared by the RGTO technique can be found in [39,40].

Resistive sensor structures based on thin layers of SnO_2_ were also fabricated using DC reactive magnetron sputtering [42]. In this technique, SnO_2_ and Au layer formation were carried out on the Leybold Z400 Sputtering System equipped with Au and Sn targets. Process conditions were as follows: dc mode, power P = 75 W, in O_2_–Ar plasma (20% O_2_–80% Ar) from a metallic target Sn (99.995%) [42], total pressure was p_tot_ = 1 × 10^−2^ mbar, oxygen pressure p_O2_ = 2 × 10^−3^ mbar, and deposition rate v_par_ = 85 and 94 nm/min. Au layers (d = 200 nm) were formed by the electrode-deposition method, using magnetron sputtering, under the following conditions: argon pressure p_Ar_ = 8 × 10^−3^ mbar, power P = 50 W. The Au layer deposition rate was 108 nm/min.

**Figure 1:**
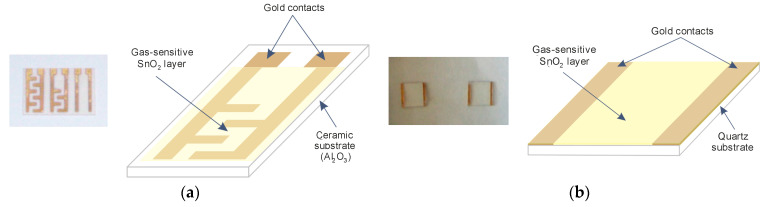
Photo and schematic of a model resistive-type sensor structure with a SnO_2_ sensor film fabricated using (**a**) RGTO or (**b**) DC reactive magnetron sputtering.

Figure 1a,b shows resistive sensing structures based on SnO_2_ thin films fabricated by RGTO technique and DC reactive magnetron sputtering. In the resistive sensing structures investigated in this work, gold electrodes were used, with the configuration shown in Figure 1a,b. The photo in Figure 1a shows three sensing structures deposited on a single ceramic substrate (Al_2_O_3_), with dimensions of 2 cm × 3 cm. These structures differ in the shape of the gold electrodes. Gold is the most commonly used electrode material in resistive gas sensors because it provides the lowest contact resistance while exhibiting poor catalytic properties compared to platinum, for example [13,22]. Figure 1a also shows a detailed schematic of one of the structures (the middle structure).

### 2.2. Measurement of Gas Response

**Figure 2:**
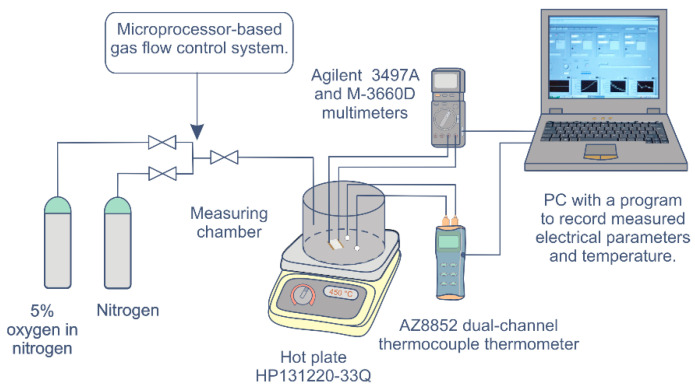
Schematic of the measuring system.

We accurately recorded the sensor structure response to changes in the gas concentration of the atmosphere through tests. The gas station consisted of an electrical power source, gas feeder, test chamber, and measuring devices. The resistance measurements of MS-SnO_2_ films were performed with dry (3% humidity) gas flow at different oxygen concentrations (1–4% of oxygen in nitrogen) whilst heating the samples to temperatures of 150–350 °C. We performed resistance measurements of RGTO-SnO_2_ layers under the dry flow gas mixture containing 2.5% of oxygen in nitrogen within a wide temperature range of 25–540 °C. Above 140 °C, we increased the temperature by 10 K every 5 min. For comparison, we carried out similar measurements of the resistance temperature dependence using a commercial Taguchi thick film (TGS 812) device. In all sensor tests, we recorded the resistance 5 min after reaching the set temperature to attain equilibrium (to establish the system resistance). We then introduced a dry gas mixture to the testing chamber. The gas feeder created constant airflow inside the test chamber, which amounted to 100 mL/min. A mass flow controller (MFC) supervised by a microprocessor system provided precise control of the gas mass flow [33]. The measurement of sensor resistance was performed using an Agilent Multimeter 3497A and a Mastech M-3660D multimeter. We controlled the temperature using a Mastech M383 multimeter and AZ8852 dual-channel thermocouple thermometer. Figure 2 shows the schematic of the measurement system.

### 2.3. Characterization of SnO_2_ Thin Layers

Qualitative X-ray phase analysis was carried out using an X-ray diffractometer (PANalytical X’Pert PRO, PANanalytical B.V., Almelo, Netherlands) with an X-ray lamp equipped with a cobalt anode at a wavelength of λ = 1.7889 Å, voltage of 30 kV, filament current of 40 mA, and angle 2θ range of 2–120°. The topography of SnO_2_ thin films deposited on a quartz substrate was investigated at a 5–20 kV accelerating voltage using a ZEISS SUPRA 25 high-resolution scanning electron microscope equipped with a TRIDENT XM4 X-ray detector for the detection of scattered radiation by EDAX. Surface analysis of SnO_2_ thin films (evaluation of surface roughness, granularity measurements, depth, and size analysis of pores) was conducted using an atomic force microscope (Digital Instruments Nanoscope). Measurements were performed at atmospheric pressure and room temperature. A high-resolution Scanning Auger Microprobe—Microlab 350 (Thermo VG Scientific)—and SAM PHI 600 model (Physical Electronics) equipped with an Ar^+^ ion gun for sample sputtering were used to conduct qualitative and quantitative analysis of the chemical composition and to estimate the thickness of the tin dioxide films. An Ar^+^ ion gun was used to measure the composition profiles of the oxide layer. Discontinuous sputtering was used to gradually remove the SnO_2_ layer. Sputtering parameters were ion energy 3 keV, beam current 1.3 µA, and crater size 2 mm × 2 mm. The Auger spectra were recorded after each sputtering period at E_p_ = 10 keV in 1.0 eV steps with a dwell time of 100 ms.

The appropriate sensitivity factors from the Thermo VG Scientific peak database editor for the elemental components were used to convert the Auger signals into atomic percentages (at %). The sputtering rate of the oxide layers removed by Ar^+^ ions was roughly estimated by ion etching a Ta_2_O_5_ film of known thickness (30 nm) grown by anodic oxidation on a Ta substrate [43]. The estimated sputtering rate was 0.2 nm/s. An Avantage-based data system was used for data acquisition and processing. The ion energy (SAM PHI 600) was 1 keV, and the sputter rate was about 1.5 nm/min (determined by ion etching of a silica sample covered by a SiO_2_ film with known thickness of 100 nm). The entire sputtering process of RGTO-SnO_2_ layers took about 5 h (in the near-surface region, the sputtering cycle stayed at 6 s, whereas it was 3 min for the bulk, and the raster area amounted to 4 mm^2^). The primary electron energy was 5 keV, and the sample current was 10 nA. The peak-to-peak signal intensity was determined from differentiated AES spectra. On this basis, the relative concentrations of the constituents were estimated using atomic sensitivity factors taken from [44]. The chemical composition of the sample surfaces was characterized by X-ray photoelectron spectroscopy (Microlab 350) using Al_Kα_ non-monochromated radiation (hν = 1486.6 eV; 300 W) as the excitation source. The pressure during analysis was 5.0 × 10^−9^ mbar. All survey spectra of the SnO_2_ surface were recorded using 150 eV pass energy. The binding energy of the target elements (Sn 3d, C 1s, O 1s, Al 2s, Au 4f, and Cl 2p) were determined with a pass energy of 40 eV at 0.83 eV resolution using the binding energy of an adventitious carbon (C 1s: 285 eV) as a reference. A linear or Shirley background subtraction [45] was applied to obtain XPS signal intensity. Peaks were fitted using an asymmetric Gaussian/Lorentzian mixed function. For data acquisition and processing, an Avantage-based data system was used.

## 3. Results and Discussion

### 3.1. X-ray Diffractometry

The investigation of SnO_2_ layers deposited on quartz substrate using DC reactive magnetron sputtering and on Al_2_O_3_ substrate by the RGTO technique was carried out in a goniometric system using a strip detector. The thickness of the layers ranged from 100 to 500 nm. Due to a large number of reflections from the substrate material during sample testing, a stable angle of incidence (α = 1.5) of the primary X-ray beam was employed. This was achieved using a parallel beam collimator in front of the proportional detector to obtain information primarily from the surface layer of the sample. Due to the possibility of recording a diffraction pattern of a beam falling on the sample surface at low angles, diffraction patterns of thin layers could be obtained by increasing the volume of diffraction material.

**Figure 3:**
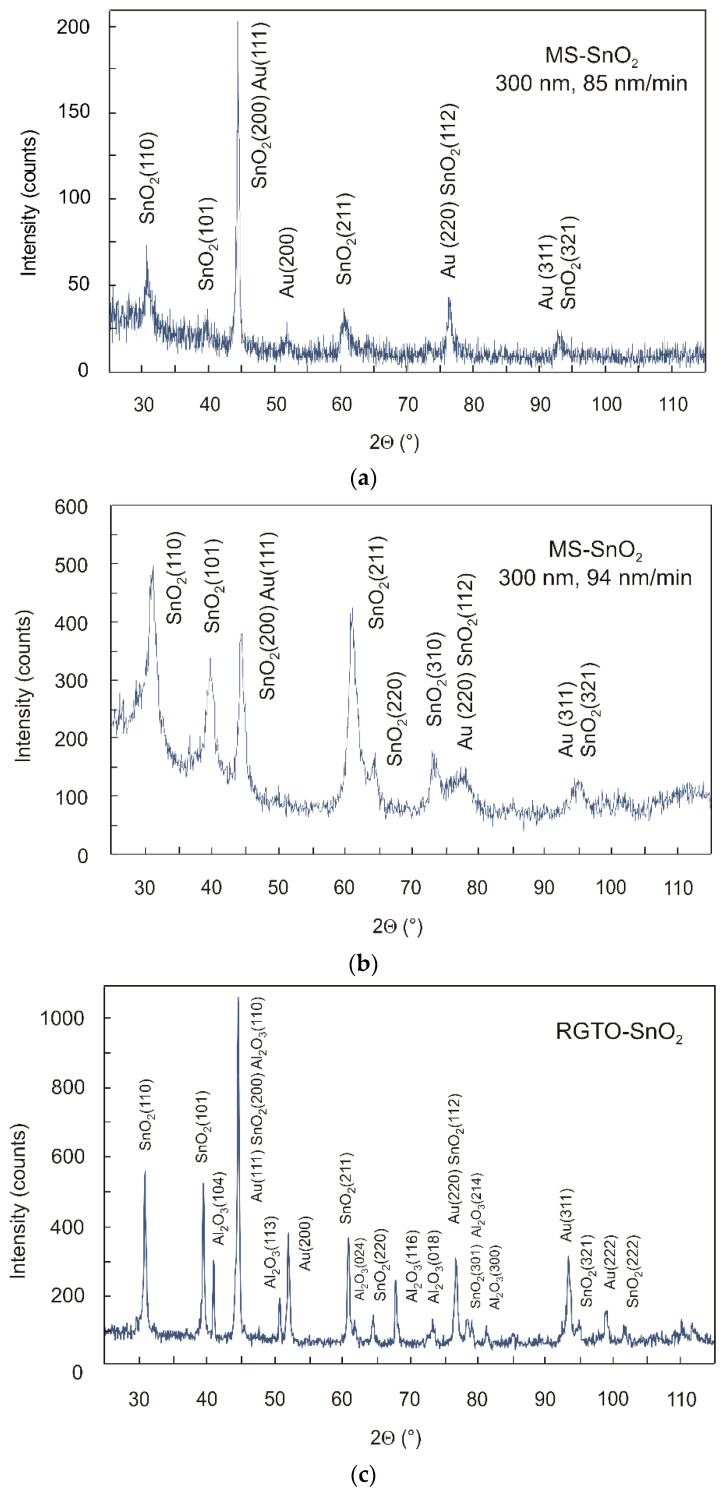
X-ray diffraction spectra for MS-SnO_2_ 300 nm thick film deposited on quartz substrate using magnetron sputtering with different deposition rate: (**a**) 85 nm/min and (**b**) 94 nm/min [48] and for (**c**) RGTO-SnO_2_ film deposited on a ceramic substrate (Al_2_O_3_-96%) with a constant angle of incidence.

With the help of the JCPDS (International Centre for Diffraction Data [46]), the analysis revealed the presence of SnO_2_ reflections (tetragonal grid, JCPDS card no. 41-1445) from the layer and Au reflections (regular grid, JCPDS card no. 04-0784). The use of samples (test structures) with gold electrodes deposited on the SnO_2_ layer caused reflections from gold in the diffraction patterns. The research also revealed the presence of Al_2_O_3_ (rhombohedral grid, JCPDS card no. 46-1212) reflections originating from the substrate material of the RGTO-SnO_2_ film (Figure 3c).

The X-ray diffraction pattern of the MS-SnO_2_ films with a thickness of 300 nm, prepared on quartz substrate by magnetron sputtering with different film deposition rates, are shown in Figure 3a,b. The results of the qualitative X-ray phase analysis revealed that the crystalline SnO_2_ layer was deposited on quartz (Figure 3a,b) or on alundum ceramics (Figure 3c), which was demonstrated by the identification of reflections originating from crystallographic planes (110), (101), (200), (211), (112), and (321). No Sn reflections were observed. In Figure 3a,b, one may notice that the preferred orientation of MS-SnO_2_ (211) became more intense when the deposition rate was slightly increased. Au reflections originating from planes (111), (200), (220), and (311) were also identified. Their occurrence was related to the sample geometry and the distance between the gold electrodes. Despite using a 1 mm inlet gap, it was impossible to eliminate that component with the measurement parameters that were used. The measurement of the crystallite size was based on diffraction patterns obtained using Scherrer’s formula and SnO_2_ reflections from the (110) plane using the geometry of the constant angle of incidence [47]. Results showed that the average size of crystallites in the 300 nm thick MS-SnO_2_ films was about 40 nm when the deposition rate was 85 nm/min and about 25 nm for deposition rate = 94 nm/min. As a result of previous calculations, we found that the average size of crystallites in the 100–500 nm thick samples was in the range of 15–40 nm [48]. The RGTO-SnO_2_ layer was formed by crystallites of about 90 nm average size. Measurement errors for the data, mentioned above, were about 10%.

### 3.2. Atomic Force (AFM) and Scanning Electron (SEM) Microscopy

**Figure 4:**
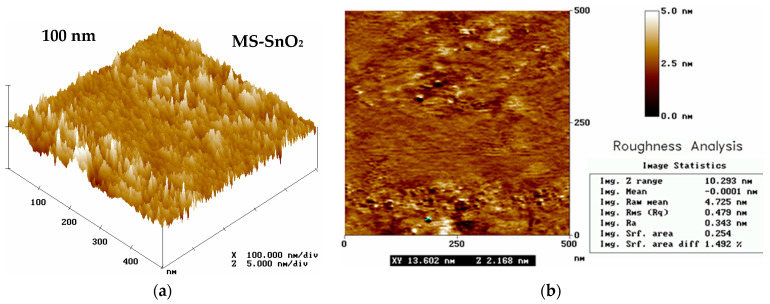
AFM images (500 nm × 500 nm) of the MS-SnO_2_ film of thickness d = 100 nm formed using the magnetron sputtering method with measurements of crystallites and film topography: (**a**) 3D image and (**b**) 2D image.

Figure 4 shows AFM images of MS-SnO_2_ film spread over a quartz substrate using magnetron sputtering. The presented surface was highly homogenous and smooth, which was consistent with the results of AFM and SEM tests given in [37,49]. The 100 nm film was very smooth, and irregularities were only 3–5 nm with an RMS of about 0.5–0.8 nm and an average grain diameter of 13 nm (Figure 4b). We described the topography of the 500 nm film in [48], with grains 124 nm long and 55 nm wide visible, and the surface irregularity varied from 4 to 12 nm with an RMS roughness of 1.5–2.4 nm.

The high smoothness and homogeneity of the SnO_2_ layer surface produced by magnetron sputtering was in accordance with results reported in [37,38,49,50], where the crystal sizes varied from 15 to 200 nm. The results of the AFM analysis revealed that the SnO_2_ films obtained by means of magnetron sputtering had a granular structure. These films were composed of similarly sized grains, which were responsible for the significant smoothness of the film surface (a lack of porosity and smaller surface development in comparison to films obtained using the RGTO technique [39,40,51]).

**Figure 5:**
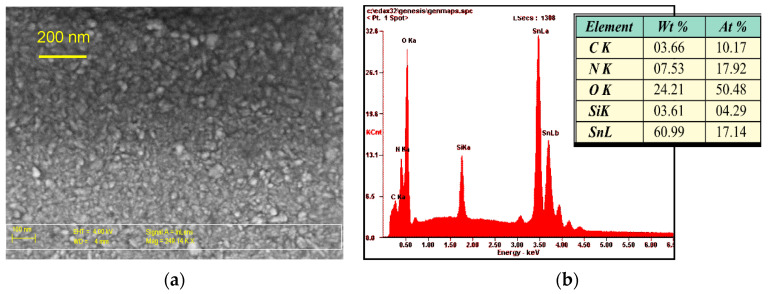
(**a**) SEM image of the MS-SnO_2_ film surface (250,000×) of thickness d = 300 nm and (**b**) results of (SnO_2_) microanalysis (qualitative and quantitative analysis).

**Figure 6:**
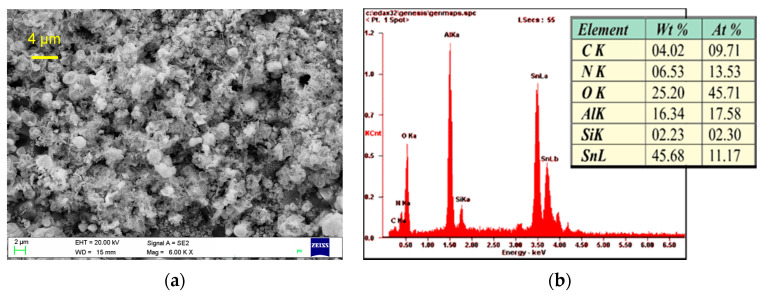
(**a**) SEM image of the RGTO-SnO_2_ film surface (6000×) (Sn deposited on alumina substrate at 270 °C) and (**b**) results of (SnO_2_) microanalysis (qualitative and quantitative analysis).

The topographies of the MS-SnO_2_ and RGTO-SnO_2_ layers are presented in Figure 5 and Figure 6. A high-resolution scanning electron microscope equipped with a scattered X-ray detector was used for an EDX local chemical analysis in selected microregions. Figure 5a and Figure 6a show SEM micrographs of the SnO_2_ thin layers produced by the magnetron sputtering and formed using the RGTO technique (Sn deposited on a ceramic substrate (Al_2_O_3_-96%) at a temperature of 543 K). The results of this qualitative and quantitative analysis are shown in Figure 5b and Figure 6b. Both surfaces differed significantly in smoothness and grain size. The SEM image (see Figure 5a) showed that the MS-SnO_2_ layer of thickness d = 300 nm was composed of grains with a diameter ranging from several nanometers to about 50 nm. They were characterized by significant surface smoothness, which results in a larger uniformity of the layer thickness and, thus, less development of the surface than the layers obtained with the RGTO technique. In contrast to the MS-SnO_2_ film, it was evident that the RGTO-SnO_2_ film (in Figure 6) was characterized by a more porous structure. SEM images also showed that layer-forming grain diameters varied from 40 to 1800 nm. In these films, grains were not closely packed together on the substrate and the layer of microholes was visible and, therefore, the sensitive area was well developed.

SEM and AFM analyses acknowledge the existence of conductive “bridges” during tin oxidation in the RGTO process that contributed to the SnO_2_ film sensitivity, reported previously by other authors [52]. Such facts are significant because the thickness and porosity of layers strongly influence toxic and explosive gas sensing abilities.

### 3.3. Auger Electron Spectroscopy (AES)

**Figure 7:**
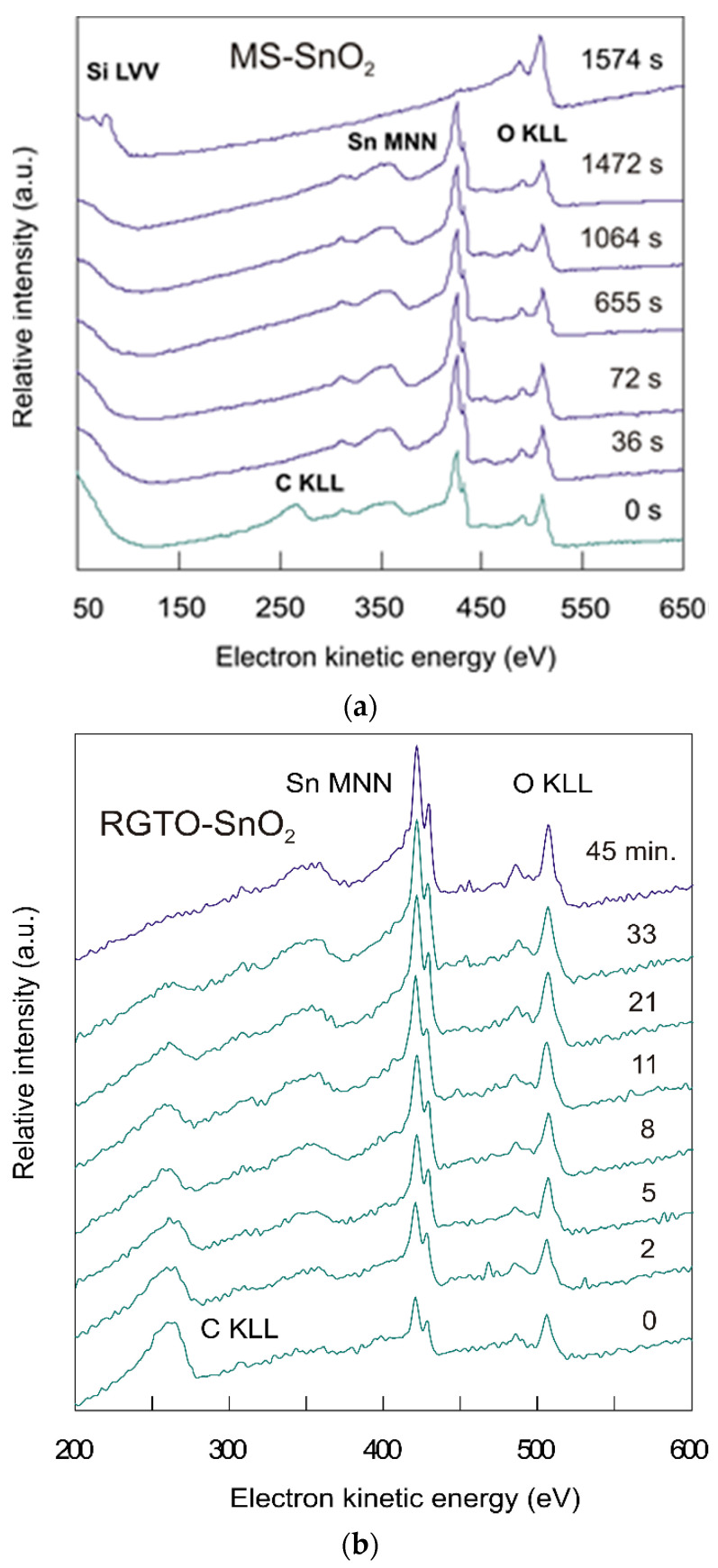
Integral Auger spectra (N(E) = f(E_kin_)) for MS-SnO_2_ sample measured (**a**) during etching of the tin dioxide layer from the outermost surface to silicon dioxide substrate and for RGTO-SnO_2_ sample (**b**) in the near-surface region.

To better understand the chemical composition of the MS-SnO_2_ and RGTO-SnO_2_ layers, surface analytical measurements and Auger electron spectroscopy combined with Ar^+^ sputtering were used. Auger spectra, registered in layer etching time, are described in Figure 7. Elements were identified using the handbook of standard Auger spectra and Avantage Peak Database Editor (Thermo VG Scientific) [53].

Figure 7a shows examples of survey spectra for the MS-SnO_2_ sample. The Sn (MNN) stable intensity (424.5 eV) and O (KLL) (511 eV) signals were clearly visible during sputtering, reflecting analytical results at various depths of the sample within the tin oxide layer. After sputtering the SnO_2_ layer away, the substrate spectra revealed the presence of silicon Si (LVV) (78 eV) and oxygen O (KLL) (507 eV). The layer thickness was about 300 nm.

For the RGTO-SnO_2_ film, the major oxygen peak was located at the energy of 506.5 eV, and there was a minor peak at 484 eV (Figure 7b). The series of peaks from 315.0 to 464.0 eV were characteristic of tin, which had its highest peaks at approximately 420.5 eV. The carbon peak was located at 263 eV. To calculate the atomic concentration of elements in the MS-SnO_2_ film, the Auger peak area (after background removal) was used. Sensitivity factors for carbon, tin, and oxygen were 0.555, 0.214, and 0.15, respectively. For the MS-SnO_2_ film, the ratio of oxygen to tin ((O)/(Sn)) concentrations was calculated using integral AES spectra. This ratio varied from 2.11 on the surface to 1.83 in depth. For the RGTO-SnO_2_ film, we estimated the ratio of (O)/(Sn) from the quotient of the Auger peak-to-peak heights of the O (KLL) signal at an electron kinetic energy of 508.5 eV, and of the tin signal at 423.5 eV corresponding to the low-energy Sn (MNN) transition using the atomic sensitivity factors (carbon: 0.14; tin: 0.9; oxygen: 0.4) taken from [44]. The (O)/(Sn) average value varied from 1.25 (in the bulk of the sample) to 1.58 (at the surface) for the weakly oxidized sample. Moreover, the locations of the most distinctive twin peaks for the RGTO-SnO_2_ films at 423.5 and 431.0 eV in differentiated spectra (430 and 437 eV for pure metallic tin [44]) indicated that tin was mainly present in an oxide form. The good separation of the doublet signal (7.5 eV) showed that either no metallic tin was present, or it was present in a small amount [54].

**Figure 8:**
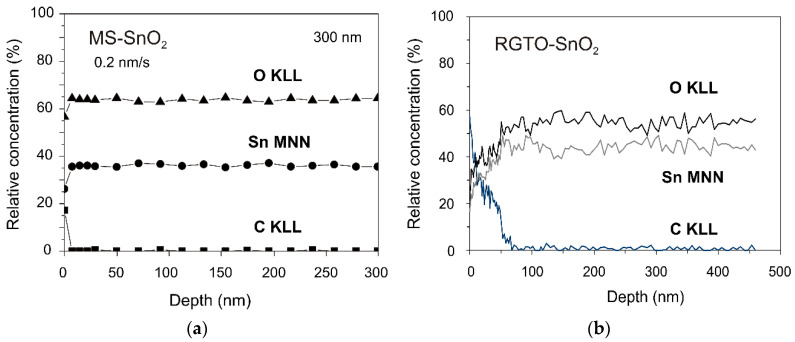
Composition depth profiles of the relative element concentration in the near-surface region and the bulk of the SnO_2_ layer formed by (**a**) magnetron sputtering and (**b**) RGTO methods.

The calculated in-depth profiles of the relative concentration of elements (Figure 8) showed that the MS-SnO_2_ and RGTO-SnO_2_ films were relatively homogenous in terms of chemical composition, except for a thin near-surface layer with a thickness of about 7 and 55 nm, respectively. Results of prior research on SnO_2_ thin layers produced by the RGTO technique showed that homogeneous, well-oxidized layers [55] with an (O)/(Sn) ratio of 1.89 on the surface were characterized by the repeatability of resistance measurements, though carbon contamination on the surface reached 50 nm in depth [33]. A relatively high depth of carbon penetration was caused primarily by the roughness of the crystal grain structure.

### 3.4. X-ray Photoelectron Spectroscopy (XPS)

**Figure 9:**
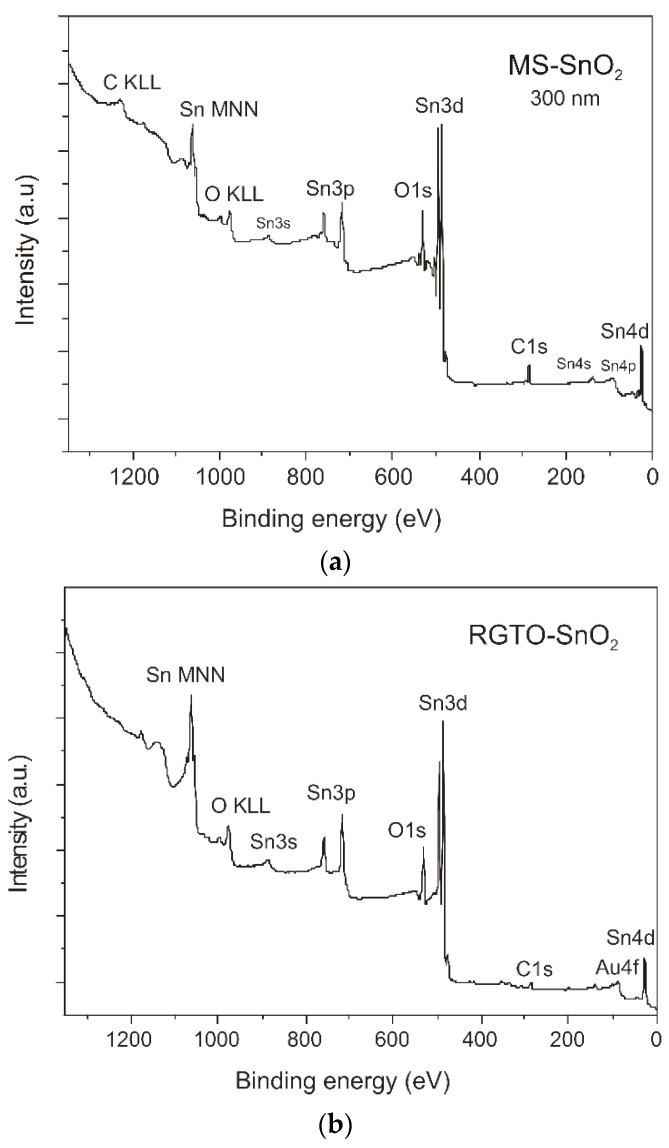
XPS survey spectra of the (**a**) MS-SnO_2_ (SnO_2_/SiO_2_) and (**b**) RGTO-SnO_2_ (SnO_2_/Al_2_O_3_) samples after 360 s ion etching.

XPS was used to analyze the chemical composition and the nature of the chemical bonds of both the MS and RGTO oxide layer surfaces. This technique provided information with a lateral resolution of ca. 2 mm × 5 mm. Figure 9 presents the XPS spectra of the SnO_2_ samples produced with different methods. From the XPS survey spectra (Figure 9a,b)), we observed signals for Sn 3d, O 1s, C 1s, Sn 3s, Sn 3p, Sn 4d, Sn 4p, and Sn 4s. Additionally, weak XPS signals at around 84, 120, and 200 eV were detected for Au 4f, Al 2s, and Cl 2p, respectively (Figure 9b). The recorded spectra were consistent with those reported elsewhere [51].

**Figure 10:**
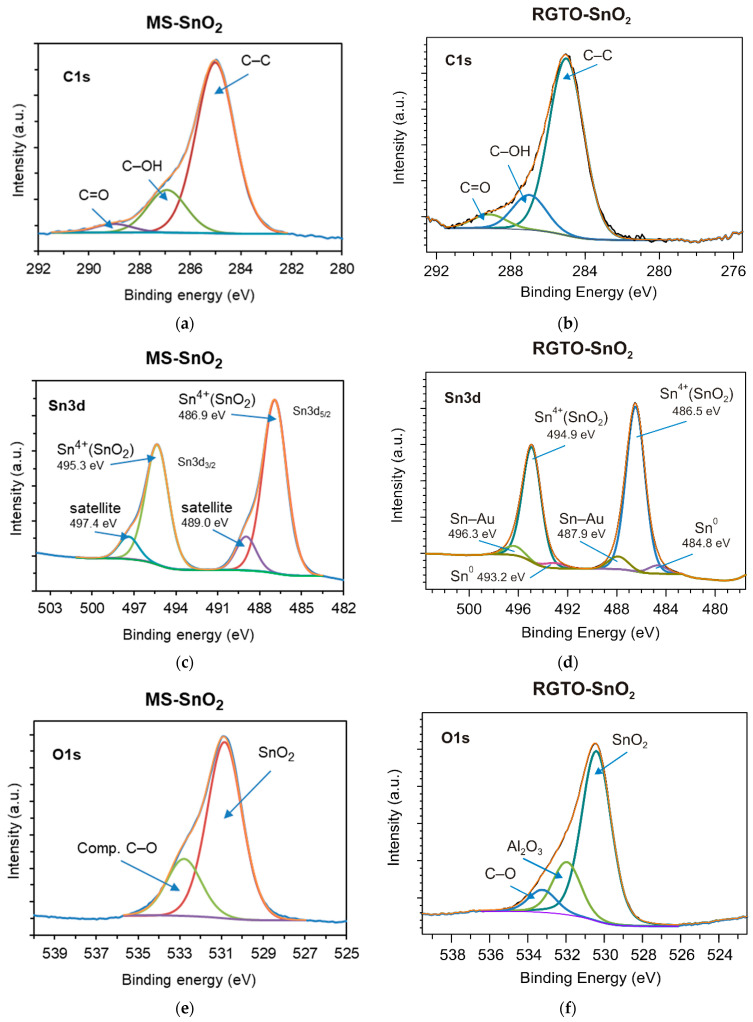
Deconvoluted (**a**,**b**) C 1s, (**c**,**d**) Sn 3d, and (**e**,**f**) O 1s core-level XPS spectra of MS-SnO_2_ (thickness = 300 nm) and RGTO-SnO_2_ films, as indicated.

Table 1 presents the binding energies of Sn 3d5, O 1s, C 1s, Al 2s, Au 4f7, and Cl 2p3 signals for both investigated samples (MS-SnO_2_ and RGTO-SnO_2_). Organic carbon contamination was observed at the outermost surface (Figure 10a,b). It consisted of a majority of hydrocarbons (C–C peak set at 285.0 eV) with a minor component of carbon species singly bonded (C–OH peak at 286.9 or 287.0 eV) or double bonded (carboxyls at 289.0 or 289.1 eV) to oxygen [56,57].

In all cases, the oxidized samples exhibited a clear Sn 3d5/2 signal at 486.9 (Ms-SnO_2_, Figure 10c) and 486.5 eV (RGTO-SnO_2_, Figure 10d). This signal was ascribed to the Sn–O bond resulting from the presence of tin dioxide [58,59,60]. However, the deconvolution of the O 1s signals suggested that two contributions may have been assigned to metal oxides 530.9 eV (MS-SnO_2_), 530.4 eV (RGTO-SnO_2_), and organic carbon contaminations (Figure 10e,f and Table 1). This finding was in accordance with the data reported by other authors [59,61,62]. Figure 10c also shows satellite peaks of Sn 3d_5/2_ and Sn 3d_3/2_ at 489.0 and 497.4 eV, respectively. Furthermore, metallic tin (Sn^0^) was present in small amounts only in the RGTO-SnO_2_ sample (Figure 10d).

**Figure 11:**
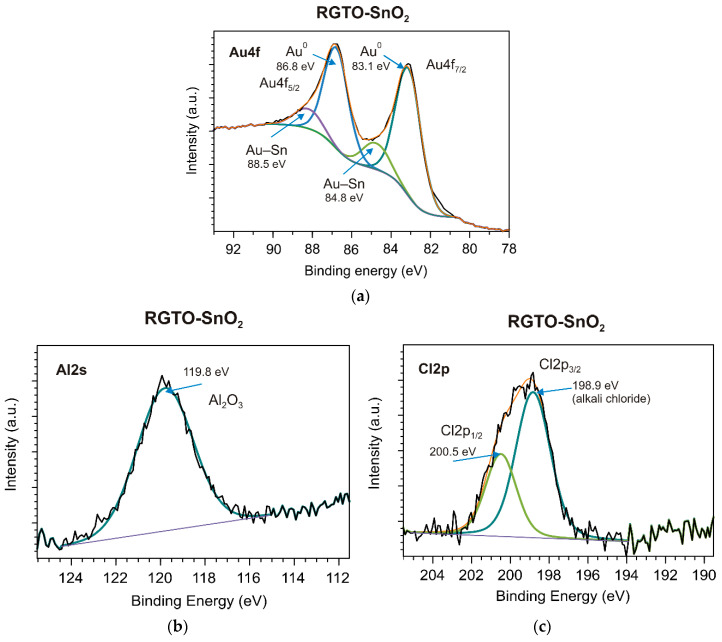
(**a**) Au 4f, (**b**) Al 2s, and (**c**) Cl 2p core-level XPS spectra of the RGTO-SnO_2_ film.

The atomic ratio of oxygen and tin (the corresponding data can be found in Table 1) indicated that the MS-SnO_2_ film ((O)/(Sn) = 2.18 at the surface and 1.89 in bulk) was more thoroughly oxidized than the RGTO-SnO_2_ film ((O)/(Sn) = 1.77 in the near-surface region). The calculated values of chemical shifts, equal to 1.9 eV [63] and 1.5 eV, respectively (with respect to pure tin, Sn 3d_5/2_ binding energy = 485.0 eV [58]), confirmed these conclusions. The Au 4f high-resolution XPS spectrum (RGTO-SnO_2_ layer) could be deconvoluted into two components (Figure 11a). The bands at 83.1 and 84.8 eV indicated the presence of metallic gold and likely gold–tin bonds [58], respectively. Note that the Au^0^ 4f_7/2_ negative binding energy shift (−0.9 eV), compared with that of the bulk metallic Au^0^ (84.0 eV), indicated interactions between Au and SnO_2_ [64]. Additionally, aluminum oxide (Al 2s binding energy = 119.8 eV) and alkali chloride (Cl 2p_3/2_ binding energy = 198.9 eV) from the substrate were also observed (Figure 11b,c) [58].

### 3.5. Gas Sensing Properties

**Figure 12:**
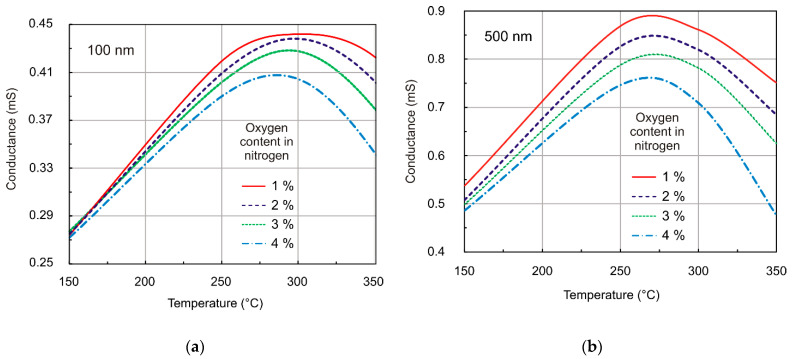
Measured conductance versus temperature of the MS-SnO_2_ thin film sensor for different oxygen content in nitrogen (magnetron sputtering deposition rate = 85 nm/min) for layer thickness of (**a**) 100 nm and (**b**) 500 nm.

Figure 12 presents the relationship between calculated conductance from the resistance measurements as a function of the temperature for various partial pressures of gas. From the measurements, we concluded that the response of the structure to the investigated gas depended on its concentration, working temperature, and film thickness. The maximum conductance was obtained at about 573 K in the 100 nm film (Figure 12a), whereas in the case of the 500 nm film, maximum conductance was reached at a lower temperature (Figure 12b). Conductance decreased above 473 K with increased oxygen pressure in a gas atmosphere for both 100 and 500 nm films.

**Figure 13:**
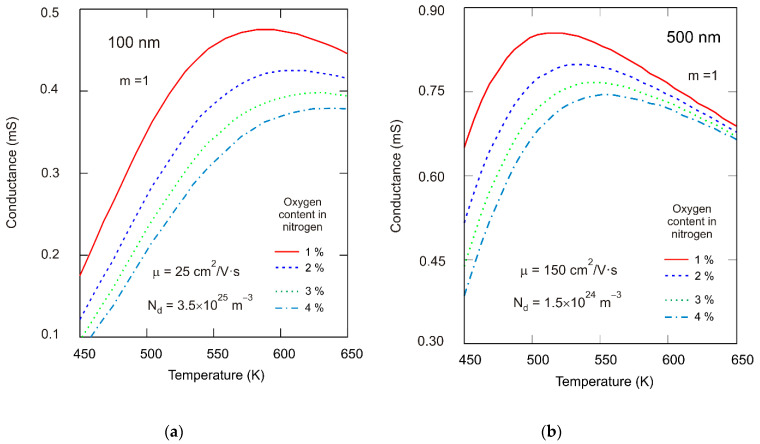
Calculated conductance versus temperature of the SnO_2_ thin film sensor for different oxygen content in nitrogen for sample thickness of (**a**) 100 nm and (**b**) 500 nm for single donors m = 1 (N_d_—donor concentration in bulk; μ—mobility of electrons—which were assumed).

Similar relationships—preferable when considering gas sensor applications—were obtained from a theoretical analysis substantiated in our previous publication [65]. The response of the resistive SnO_2_ film to the action of oxidizing gas was heavily dependent on the one hand, on the mechanism of the interaction between gas molecules and the SnO_2_ film, and on the other hand, on the transport of carriers in the depleted region induced by a negative charge trapped by the adsorptive acceptor-type surface states. The surface space charge region, induced by adsorbed oxygen ions, contributed significantly to the conductance of the semiconducting SnO_2_ layers. Figure 13 shows the results of theoretical calculations of the relationship between conductance and temperature based on the assumption that the 100 and 500 nm films were totally depleted, and donors (oxygen vacancies) in the bulk were mobile [66]. Measurements and calculations were carried out for identical partial pressures of oxygen. Comparing Figure 12 and Figure 13, we can conclude that the resulting values of conductance per square were in line with the measurement data. As suggested by theoretical analysis, the observed conductivity maximum may be linked to the minimal oxygen ion coverage at a given temperature (equalization of coverage by different oxygen ions), which was strongly related to the layer structure (volume doping level). Since structural tests revealed the crystalline structure of grains, findings from the numerical analysis may be used to interpret measured characteristics of the investigated films. For a layer with a given thickness, for example, 100 nm, the calculations showed that the maximum conductance shifted towards lower temperatures as the partial pressure of oxygen decreased or when the donor concentration (N_d_) in bulk increased [65]. Experimentally, the maximum conductance was observed at 450 K for SnO_2_ nanocrystalline layers with a grain diameter of <20 nm. Additionally, maximum conductance was observed between 550 and 600 K for thin polycrystalline layers with a thickness less than 500 nm and an average grain size of 15 nm [67].

**Figure 14:**
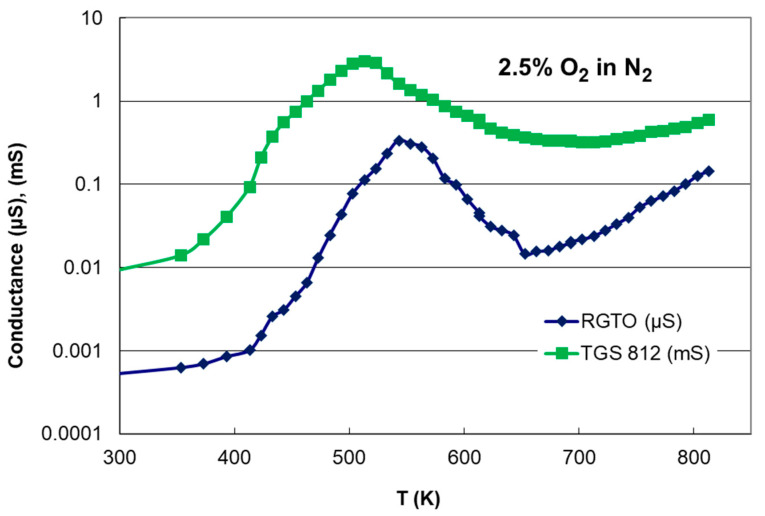
Measured conductance versus temperature of the RGTO-SnO_2_ thin film sensor (Sn deposited at a temperature of 255 °C) and the thick film (TGS812) sensor. The heating rates were 2 K/min for 2.5% oxygen concentration in nitrogen.

The measured dependencies of conductance changed as a function of temperature for 2.5% oxygen concentration in nitrogen, a RGTO-SnO_2_ thin-film sensor (with tin deposition temperature of 255 °C), and a commercially available thick-film sensor TGS 812 are summarized in Figure 14. We could see that both the n-type sensor devices exhibited a similar conductance behavior versus temperature (in the range of 25–540 °C), with a specific maximum. We obtained a conductance maximum of about 543 and 523 K for the RGTO-SnO_2_ sensor and thick-film sensor TGS 812, respectively.

Previously, we provided our interpretation of conductance measurement results for other O_2_ concentrations (4%, 21%, 75%) in nitrogen and tin deposited at 270 ℃ [33], in which appropriate potential chemical reactions responsible for the sensor response changes were discussed in detail.

The method used to produce thin-film layers determined their layer thickness, working temperature, shape, and grain size. These different structures had different responses to gases. For example, as seen in our previous publication [33], sensor structures obtained with the RGTO technique had maximum sensitivity to oxygen in the researched atmosphere in the temperature range of 533–558 K. Other investigations in the literature have shown maximum conductance at about 590 K in the case of thin films [67] and at lower temperatures (555 K) for thick films [68].

Minimum resistance (maximum conductance) at the desired temperature is a very positive phenomenon used in gas sensors. At optimal temperature, the sensor response is independent of small changes in temperature, which allows the requirement for a precise temperature stabilizer to be relaxed [48].

## 4. Conclusions

The results of the qualitative X-ray phase analysis revealed that the crystalline SnO_2_ layer was deposited on quartz or on alundum ceramics. The results of AFM revealed the granular structure of the SnO_2_ films produced using DC reactive magnetron sputtering. These films were composed of similarly sized grains, which were responsible for significant smoothness of the film surface, i.e., the lack of porosity and a smaller surface development, in comparison to films produced using the RGTO technique.

The gas molecule adsorption mechanism on the surface of the oxide semiconductor was determined mainly by the chemical and electron properties of the surface and near-surface region. Electric properties of SnO_2_ thin layers strongly depended on their stoichiometry and microstructure [13]. The (O)/(Sn) value, calculated using the integral AES spectrum, changed from 1.58 at the surface to 1.25 in the layered bulk RGTO-SnO_2_ and from 2.11 to 1.83 in MS-SnO_2_. We estimated the depth of the carbon presence at around 50 nm for RGTO-SnO_2_ and 7 nm for MS-SnO_2_. The structure response to gas action was dependent on the working temperature and film thickness. In particular, maximum conductance was observed at about 573 K in the case of the 100 nm film, whereas in the case of the 500 nm film, the maximum conductance was observed at a lower temperature. The maximum for the temperature characteristics of conductance (for similar concentration levels of oxygen in nitrogen), was also obtained from a theoretical analysis of oxygen adsorption on the surface of SnO_2_ (110) for totally depleted 100 and 500 nm layers. For the investigated films, there was agreement between the measurements and calculations for the temperature dependence of conductance.

The structural, chemical (XRD, SEM, AFM, AES, XPS), and sensor (measurements of changes in resistance in the gas atmosphere) tests enabled us to determine that parameters such as the layer structure, surface morphology, working temperature, and gas concentration were key factors for the optimum operation of the SnO_2_ thin film sensor in an oxygen atmosphere.

## Figures and Tables

**Table 1:** XPS study of the chemical state and chemical composition of the surface of the sensitive layers SnO_2_ obtained using (a) magnetron sputtering method and (b) RGTO technique (Sn deposited at 270 °C).

Layer	Sn3d5 (eV)	C1s (eV)	O1s (eV)	Al2s (eV)	Au4f7 (eV)	Cl2p3 (eV)	Chemical State	Chemical Composition at (%)
(a) SnO_2_/ SiO_2_	486.9 - - -	- 285.0 286.9 289.0	530.9 - 532.8 -	- - - -	- - - -	- - - -	SnO_2_ C–C comp. (C, O, H) organic comp. (C, O)	O (46.4) C (32.3) Sn (21.3)
(b) SnO_2_/ Al_2_O_3_	486.5 - - - - - - - - 484.8 487.9	- 285.0 - 287.0 289.1 - - - - - -	530.4 - 532.0 533.2 - - - - - - -	- - - - - 119.8 - - - - -	- - - - - - - 83.1 84.8 - -	- - - - - - 198.9 - - - -	SnO_2_ C–C metallic oxide (Al, O) comp. (C, O, H) organic comp. (C, O) Al with O (Al_2_O_3_) alkali chloride Au Au with Sn Sn^0^ Sn–Au	O (44.9) Sn (25.3) C (21.5) Al (5.2) Au (1.6) Cl (1.5)

## Data Availability

Not applicable.
